# Diffusion of Phthisis

**Published:** 1904-09-10

**Authors:** 


### The Hospital.

A Journal of

The Medical Sciences and Hospital Administration.

Vol. XXXVI.—No. 937. SEPTEMBER 10, 1904.

### "DIFFUSION OF PHTHISIS."

A letter under the above title, which has appeared in the columns of a non-medical contemporary, over the signature of that skilful *littérateur* and ingenious writer of fiction, the Rev. S. Baring-Gould, seems to afford an illustration of popular notions concerning the conveyance of infection and the propagation of disease of such a kind as not to be undeserving of comment. The writer tells us that, "in 1890," a labouring man in his parish died of consumption, that his son, a fine able-bodied young man, who has slept in the room in which his father died, is "now" in a decline, and that he (the writer) can "scarcely doubt" but that this is due to the "germs having remained in the room." He proceeds to comment adversely upon the sanitary authorities of Tavistock for having informed him that, as phthisis is not on the list of notifiable diseases, they are unable to disinfect either the house generally or the room which the son has occupied, except at the applicant's expense; and he expresses his conviction that, "when consumption is so prevalent a malady and carries off so many victims, the sanitary authorities should do their utmost to check its ravages." He brings forward, as a proof that the disease is spread by "germs," the interesting piece of evidence that, at Davos, since the place has become a resort for invalids suffering from phthisis, "the villagers are now attacked by it"; and he tells us that, while the question of disinfection was still pending, he had the son "removed to a hospital," where, presumably, he would be in a position to communicate "germs," in a state of comparative freshness and activity, to the occupants of other beds in his vicinity.

Admitting, in the first place, that the evidence in proof of the communication of phthisis by the bacillus of tubercle is overwhelming, and that it in no way requires to be strengthened by the misfortunes of the villagers in and around Davos, we cannot but think that a belief in the passive survival of the "germs" in a particular bedroom for 14 years, to be followed by a sudden display of their activity in the person of the son of the first patient, a son who had probably been occupying the room with impunity for the whole of the intervening period, and who must probably have been exposed to many other sources of infection, is one which seems to arise less from knowledge than from something much akin to superstition; while the touching faith displayed in the efficacy of "disinfection" is of an almost similar character. A country clergyman, not only of quite exceptional ability and intelligence, but who is shown by his writings to be in very complete sympathy with his flock, able to understand their feelings and to penetrate to the very lowest strata of their minds, might not unreasonably be expected to know, and even to preach, that the most effectual disinfectants as against the tubercle bacillus are fresh air and sunlight. It is a pity that, on the death of his old parishioner 14 years ago, no one taught the survivors to scrub their floors and to set their doors and windows wide open. In the light of modern knowledge it is certain that such teaching, proceeding from the lips of a clergyman, would not only have been more efficacious than any similar advice from the doctor, but that it would also have been more efficacious than anything which could have been done by the sanitary authorities, in whose defence it may at least be said that, even if they had "disinfected" the bedroom 14 years ago, certain bacilli might, in the course of the long interval, have found means of reoccupying it.

It is in the power of every discreet and sympathetic country clergyman, by precept and example, to bring about improvements in the sanitary state of his parish, to obtain the removal of nuisances, the drainage of damp places, the adoption of at least comparative cleanliness, the better ventilation of dwellings, and, to sum up the matter in a sentence, to place many difficulties in the way of a 14-year survival of pathogenetic bacilli. When everything possible has been done by personal effort in these directions, it will be soon enough to think of calling upon Hercules, or to find fault with "sanitary authorities" whose inaction, presuming it to have been blameworthy, will at least have been only a reflection of the public opinion of their constituency. We would venture to suggest to the clergy the devotion of some portion of their leisure to the business of taking counsel with the parish doctor, and of inquiring from him what are the conditions and the practices most hurtful to the health of the labouring population in the vicinity. And they will not fail, we think, to hear of many hurtful influences more likely to be operative, and certainly more easy to be altered, than a single death from phthisis which occurred 14 years ago.

Nothing, of course, can be farther from our wish than any attempt to minimise the risks of the communication of tuberculous disease from one inhabitant of a cottage to others, even if we think these risks are likely to be greater during the life of the patient than fourteen years after his death. But here again we think the personal influence of the "parson" may be eminently useful. Tennyson's Northern Farmer was not much influenced by preaching:

"I thowt a said whot a owt to 'a said an' I coom'd awaäy."

And the poor, we believe, are apt to regard sanitary counsel from the doctor very much in the same light. They look upon it as something which is his official duty to say to them, and his advice to open their windows, which are often nailed up, produces no other result than a grin. But spiritual advice from the doctor and sanitary advice from the parson are far more likely to be received and acted upon. Neither one nor the other appears to be perfunctory; and rustics, although they never heard the word, have a very acute appreciation of the thing which it denotes.

